# Immature Platelet Counts and Thrombopoietin Plasma Concentrations in Thrombocytopenic and Non-thrombocytopenic Preterm Infants

**DOI:** 10.3389/fped.2021.685643

**Published:** 2021-06-25

**Authors:** Hannes Sallmon, Andreas Weimann, Christoph Bührer, Boris Metze, Christof Dame, Malte Cremer

**Affiliations:** ^1^Department of Neonatology, Charité – Universitätsmedizin Berlin, Berlin, Germany; ^2^Department of Pediatric Cardiology, Charité – Universitätsmedizin Berlin, Berlin, Germany; ^3^Department of Congenital Heart Disease/Pediatric Cardiology, Deutsches Herzzentrum Berlin (DHZB), Berlin, Germany; ^4^Labor Berlin – Charité Vivantes GmbH, Berlin, Germany

**Keywords:** megakaryopoiesis, thrombocytopenia, thrombopoiesis, very low birth weight infant, transfusion

## Abstract

**Objective:** Immature platelet counts (IPC) may prove useful in guiding platelet transfusion management in preterm neonates. However, the relationship between IPCs and thrombopoietin (Tpo) concentrations has not been evaluated in preterm neonates.

**Methods:** Prospective cohort study in thrombocytopenic (*n* = 31) and non-thrombocytopenic very low birth weight (VLBW) infants (*n* = 38), and healthy term neonates (controls; *n* = 41). Absolute platelet counts (APCs), IPCs, and Tpo concentrations were assessed by a fully-automated hematological analyzer (IPC, APC) and by ELISA (Tpo concentrations) in parallel on day 1 of life (d1), d3, and d7.

**Results:** In healthy term neonates, APCs remained stable between d1 and d3. In non-thrombocytopenic VLBW infants, APCs increased from d1 to d7, while in the thrombocytopenia group, APCs declined from d1 to d3, before they slightly increased again by d7. Median IPCs were similar in healthy term vs. non-thrombocytopenic VLBW infants and remained stable between d1 and d3 (*p* > 0.05). Notably, IPCs significantly increased between d3 and d7 in both non-thrombocytopenic and thrombocytopenic VLBW infants. However, in thrombocytopenic VLBW infants, IPC values were significantly lower at each time point as compared to non-thrombocytopenic VLBWs (*p* < 0.001). In each subgroup, Tpo concentrations increased from d1 to d3. The median Tpo concentrations were significantly higher in thrombocytopenic as compared to non-thrombocytopenic VLBW infants at d3 (*p* = 0.01) and d7 (*p* = 0.002).

**Discussion:** Term infants, thrombocytopenic, and non-thrombocytopenic preterm infants display similar developmental changes in indices of megakaryopoietic activity. In thrombocytopenic preterm infants, however, the responsive increases in Tpo and immature platelets appear to be developmentally limited.

## Introduction

Thrombocytopenia is uncommon in healthy neonates but affects up to one-third of all infants admitted to a neonatal intensive care unit. The incidence of thrombocytopenia inversely correlates with birth weight and gestational age, and up to 70 % of all preterm infants with a birth weight <1,000 g exhibit at least once an absolute platelet count (APC) <150/nl during neonatal intensive care. In very low birth weight (VLBW, <1,500 g) infants, early-onset thrombocytopenia (diagnosed within <72 h after birth) is most frequently of maternal origin (e.g., placental insufficiency, preeclampsia). In these infants, thrombocytopenia is usually moderate (50–150/nl) and thought to be caused, for example, by excessive erythropoiesis at the expense of megakaryopoiesis (1). Determination of immature platelet counts (IPC) is an innovative tool to examine the extend of platelet production in peripheral blood samples. Immature platelets are newly released platelets (<24 h old) that are larger and hemostatically more active than “older” platelets. IPC have been reported in preterm neonates and may prove useful in guiding platelet transfusion management (1, 2). However, the relationship between IPCs and thrombopoietin (Tpo) concentrations has not been systematically evaluated in preterm VLBW neonates. In order to characterize neonatal megakaryopoietic/thrombopoietic activity, we therefore prospectively investigated APCs and IPCs, as well as Tpo concentrations in VLBW infants (with and without early-onset thrombocytopenia) and in healthy term neonates.

## Methods

### Subjects

In 69 VLBW infants, blood sampling was performed at day 1 (d1), day 3 (± 1 day), and day 7 (± 2 days) after birth. VLBW infants were assigned to the thrombocytopenia group if at least one platelet count (d1, d3, and/or d7) <150/nl was detected. Thrombocytopenic infants were compared to non-thrombocytopenic VLBW infants (platelet counts >150/nl at each time point studied). In addition, both groups were also compared to 42 term neonates, if their blood count, interleukin-6, and/or C-reactive protein concentrations were within normal ranges, no antibiotic treatment was initiated, and if neonates appeared healthy upon follow-up during the first 72 h. In term infants, only d1 and d3 samples were obtained. The Institutional Review Board approved the study (EA1/229/08) and informed written parental consent was obtained.

### Absolute and Immature Platelet Counts and Thrombopoietin Concentration

Blood count parameters were measured using a SYSMEX XE-2100 (Sysmex, Kobe, Japan) blood analyzer. APCs and IPCs were reported as absolute values per nanoliter. Tpo concentrations were determined in duplicate by ELISA (R&D Systems, Minneapolis, MN, USA). The lower detection limit of Tpo was 30 pg/ml. If the Tpo concentration was below the detection limit, the value was arbitrarily set to 15 pg/ml.

### Statistical Analysis

Comparisons for demographic variables were performed by the Kruskal-Wallis Test or by chi-square test with Fisher's exact test as appropriate. Comparisons within groups between time points were performed by *post-hoc* Wilcoxon Test after Friedman's two-way analysis of variance. Comparisons between thrombocytopenic and non-thrombocytopenic VLBW infants were performed by the Mann-Whitney-*U*-test. Statistical analysis was performed using the PASW statistics software (Version 21, SPSS Inc., Chicago, IL, USA). A *p*-value of < 0.05 was considered statistically significant.

## Results

Among the 69 VLBW infants, 38 infants were diagnosed with early-onset thrombocytopenia. Thrombocytopenic infants had a significantly lower gestational age and birth weight as compared to the 31 non-thrombocytopenic VLBW infants. The frequency of intraventricular hemorrhage (IVH all stages, but also mild-to-severe IVH ≥2°) was not significantly higher in the thrombocytopenia subgroup, as compared to non-thrombocytopenic infants (Table 1).

**Table 1:** Clinical characteristics of healthy term and preterm VLBW infants with and without early-onset thrombocytopenia.

	**Healthy term infants**	**VLBW infants without thrombocytopenia**	**VLBW infants with thrombocytopenia**	*p*-value^*^
Number of patients (*n*)	41	31	38	-
Sex female (*n*, %)	21 (51%)	13 (42%)	10 (26%)	0.171
Birth weight (grams)	3,425(2,590 – 4,080)	1,200 (660 – 1,495)	780 (382 – 1,450)	**0.000**
Gestational age (weeks)	39 + 4 (37 + 1–41 + 6)	28 + 6 (23 + 5–32 + 2)	26 + 1 (23 + 2–33 + 2)	**0.001**
Apgar 5 min	10 (6–10)	8 (4–10)	7 (2–9)	**0.006**
Antenatal steroids (*n*, %)	0 (0%)	22 (71%)	23 (61%)	0.365
Surfactant (*n*, %)	0 (0%)	20 (65%)	30 (79%)	0.182
Mechanical ventilation (*n*, %)	0 (0%)	12 (39%)	30 (79%)	**0.001**
Early-onset sepsis (*n*, %)	0 (0%)	7 (23%)	15 (39%)	0.134
Intraventricular hemorrhage (*n*, %)	0 (0%)	5 (16%)	11 (29%)	0.209
Intraventricular hemorrhage ≥ grade 2 (*n*, %)	0 (0%)	1 (3%)	5 (13%)	0.145
Incidence of severe thrombocytopenia, (<50/nl; *n*, %)	0 (0%)	0 (0%)	2 (5%)	-
Platelet transfusion (during the first 7 days of life; *n*, %)	0 (0%)	0 (0%)	1 (3%)	-

*VLBW, very low birth weight infant*.

* *p-value for comparison between thrombocytopenic and non-thrombocytopenic VLBW infants*.

*Statistical significance is indicated by bold values*.

**Figure 1:**
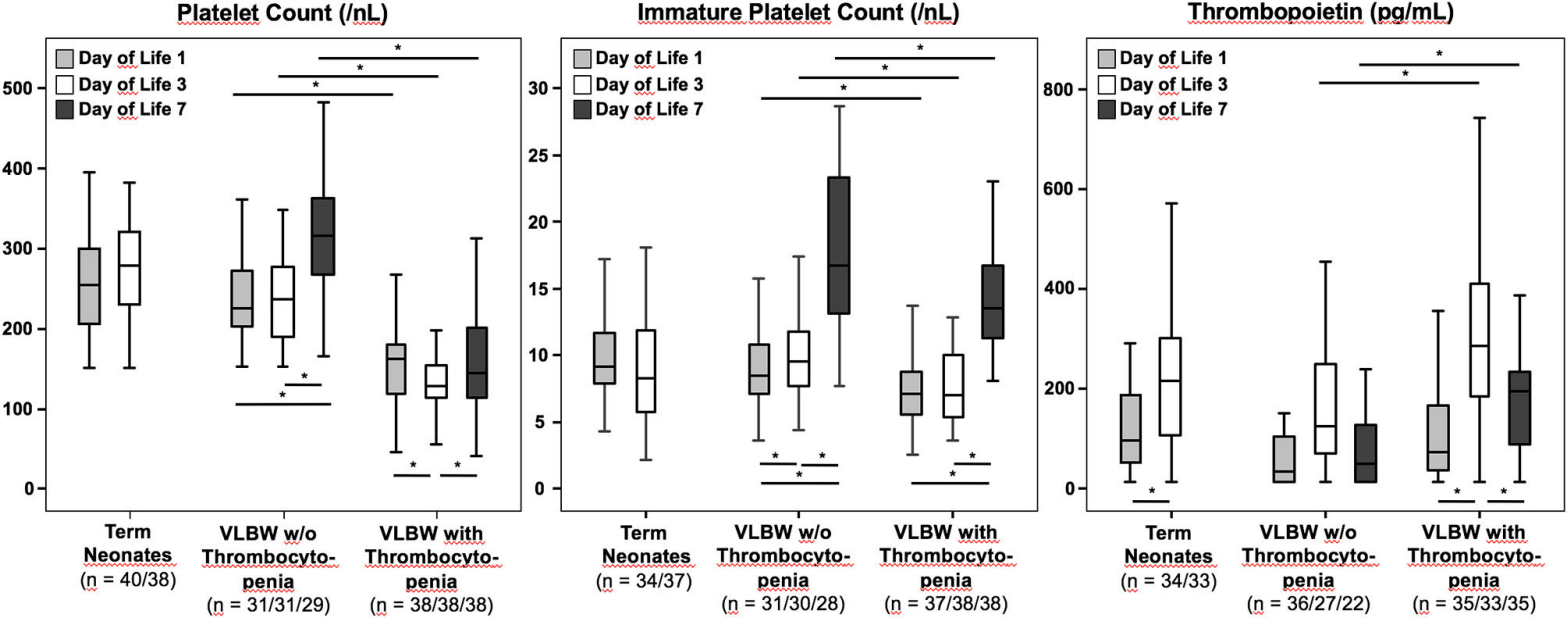
Absolute platelet counts, immature platelet counts, and thrombopoietin plasma concentrations in VLBW preterm infants (with and without thrombocytopenia) and in healthy term newborns. Data were analyzed at different time points: Day of life 1, 3, and 7 for VLBW infants, and day of life 1 and 3 for healthy term controls. The bottom of the vertical lines represents the 5th percentile, the top of the vertical lines the 95th percentile. The bottom and the top of the boxes mark the 25th and 75th percentile, respectively. The median lines mark the 50th percentile (median). Tpo concentrations could only be determined in 95/110, 93/110, and 57/69 blood samples at d1, d3, and d7, respectively, due to a lack of residual plasma in some specimens. *p < 0.05.

In healthy term neonates, APCs remained stable between d1 and d3 (d1 255/nl, d3: 280/nl). In non-thrombocytopenic VLBW infants, APCs increased from d1 to d7 (d1 225/nl, d3: 236/nl, d7: 316/nl), while in the thrombocytopenia group, APCs declined from d1 to d3, before they slightly increased again by d7 (d1 163/nl, d3: 129/nl, d7: 145/nl) (Figure 1).

The median IPC values were similar in healthy term vs. non-thrombocytopenic VLBW infants and remained stable between d1 and d3 (d1: 9.17/nl, d3: 8.27/nl vs. d1: 8.5/nl, d3: 9.6/nl; *p* > 0.05). Notably, IPC values significantly increased between d3 and d7 in both non-thrombocytopenic and thrombocytopenic VLBW infants (*p* < 0.05). However, in thrombocytopenic VLBW infants, IPC values were significantly lower at each time point as compared to non-thrombocytopenic VLBWs, in particular between d3 and d7 (d1: 7.2/nl, d3: 7.1/nl, d7: 13.5/nl vs. d1: 8.5/nl, d3: 9.6/nl, d7: 16.7/nl; *p* < 0.001).

In each subgroup, Tpo concentrations increased from d1 to d3 (*p* < 0.05). The median Tpo concentrations were significantly higher in thrombocytopenic as compared to non-thrombocytopenic VLBW infants at d3 and d7 (d1: 73 pg/ml, d3: 285 pg/ml, d7: 195 pg/ml vs. d1: 35 pg/ml, d3: 125 pg/ml, d7: 49 pg/ml; d1: *p* > 0.05, d3: *p* = 0.01, d7: *p* = 0.002).

## Discussion

To our knowledge, parallel determination of APCs, IPCs, and Tpo for non-invasive assessment of megakaryopoietic activity has not been previously assessed in VLBW infants, who are especially vulnerable to thrombocytopenia and major hemorrhage (1).

Of note, developmental differences between neonatal and adult megakaryopoiesis exist and likely contribute to the higher vulnerability of preterm infants to thrombocytopenia (1, 4). Neonates also show an impaired upregulation of their Tpo levels in response to low platelet counts. In addition, Tpo in neonatal thrombocytopenia increases megakaryocyte progenitor proliferation, but it does not promote megakaryocyte maturation (increased ploidy), thus leading to an increased number of smaller megakaryocytes (as compared to adult thrombocytopenia) (1). These developmental limitations are somewhat circumvented by the higher responsiveness of human megakaryocytes to Tpo and the prolonged half-life of neonatal platelets (4, 5).

As previously reported, circulating Tpo concentrations increase within the first 96 h in healthy term infants (3). The mechanism behind this postnatal increase is yet to be determined, but the fact that APCs and IPCs remain constant argues against the previous hypothesis that the increase of Tpo results from reduced blood volume within the first days of life. In VLBW infants with early-onset thrombocytopenia, this pattern of longitudinal Tpo concentrations is maintained with moderately higher levels (Figure 1). The longitudinal analysis also indicates that in non-thrombocytopenic VLBW infants, the d3 peak in Tpo is followed by a significant increase in IPC and APC values at d7, which is preserved in VLBW infants with mild thrombocytopenia. To which extent these responses are adequate or not should not be concluded from our study population, because only two out of 38 VLBW infants exhibited severe thrombocytopenia (APCs <50/nl), triggering platelet transfusion in one of them. Of note, thrombocytopenic infants in this study had early-onset thrombocytopenia, which was mild in almost all cases. In this type of thrombocytopenia, which is common in immature preterm infants and rarely necessitates platelet transfusions (as confirmed in this cohort), low platelet counts are usually of maternal origin (e.g., due to increased erythropoiesis at the expense of thrombopoiesis in response to preeclampsia) (5). However, we cannot exclude the possibility that differences in gestational age and underlying pathology might have influenced our results, which were obtained from only a single-center cohort of limited size. Thus, confirmatory studies in larger cohorts are warranted, which should include thrombocytopenias of different causes and severity, such as those seen in sepsis or asphyxia. We have previously reported that preterm infants with late-onset sepsis/necrotizing enterocolitis exhibit low IPC values (indicating hyporegenerative thrombocytopenia) and often require platelet transfusions (6).

Furthermore, a study in children with immune thrombocytopenia found that higher immature platelet fraction values were associated with less bleeding (5). Thus, the kinetics of IPCs in thrombocytopenic VLBW infants may not only be relevant for the recovery of normal platelet counts but also for understanding the bleeding risk. However, the potential of immature platelets to predict bleeding has not yet been assessed in the early neonatal period in VLBW infants.

In conclusion, IPCs may not only be useful to categorize neonatal thrombocytopenias, as previously suggested with emphasis on developmental patterns of immature platelet release according to gestational age and APCs (2, 6), but also for assessing the bleeding risk in neonates (7, 8). Considering the recent endeavors toward restrictive platelet transfusion policy in preterm infants (9), serial measurements of IPCs might prove useful in decision-making on when to transfuse donor platelets and, therefore, warrant further investigation.

## Data Availability Statement

The raw data supporting the conclusions of this article will be made available by the authors, without undue reservation.

## Ethics Statement

The studies involving human participants were reviewed and approved by the Charité Institutional Review Board (EA1/229/08). Written informed consent to participate in this study was provided by the participants' legal guardian/next of kin.

## Author Contributions

HS, AW, CD, and MC planned and conducted the study. HS and MC wrote the manuscript. All authors contributed to analysis and interpretation and approved the final version of the manuscript.

## Conflict of Interest

MC served on the advisory board of SYSMEX Deutschland GmbH (Hamburg, Germany). AW is employed by the company Labor Berlin – Charité Vivantes GmbH. The remaining authors declare that the research was conducted in the absence of any commercial or financial relationships that could be construed as a potential conflict of interest.
